# Social networks and loneliness differ between LGBTIA and cis-heterosexual persons: results from a two-wave survey in Germany

**DOI:** 10.1038/s41598-025-92021-9

**Published:** 2025-03-07

**Authors:** Philip Oeser, Tristan Wellendorff, Hendrik Napierala, Marie Bolster, Paul Gellert, Wolfram Herrmann

**Affiliations:** 1 Institute of General Practice and Family Medicine, Charité-Universitätsmedizin Berlin, Corporate Member of Freie Universität Berlin and Humboldt-Universität zu Berlin, Charitéplatz 1, 10117 Berlin, Germany; 2 Institute of Medical Sociology and Rehabilitation Science, Charité-Universitätsmedizin Berlin, Corporate Member of Freie Universität Berlin and Humboldt-Universität zu Berlin, Charitéplatz 1, 10117 Berlin, Germany

**Keywords:** LGBT, Social network, Emotional loneliness, Social loneliness, Mental health, Health services, Risk factors

## Abstract

Current research suggests LGBTIA persons to be lonelier than cis-heterosexual persons. While they rely more on friends than on family as support network, their social network and weekly contact to family and friends, as well as the association between the social network and loneliness have not been fully explored yet. The aim of this analysis was to examine differences in the social network between LGBTIA and cis-heterosexual persons in Germany, and how these differences affect loneliness. Data was collected through an online survey conducted in two independent waves in March/April 2020 and in January/February 2021. Linear regression analyses were performed to examine the influence of the social network on loneliness. Of 6784 participants, 5442 identified as LGBTIA. Weekly contact to family was lower in the LGBTIA group than in cis-heterosexuals. LGBTIA were less likely to be in a relationship. Identifying as LGBTIA increased social and emotional loneliness. Differences in social network partly explained the risk for social loneliness of LGBTIA persons and, to a lower degree, the risk for emotional loneliness. We encourage health care professionals to inquire about sexual orientation, gender identity, and relationship status to raise awareness for feelings of loneliness and related health problems.

## Introduction

Lesbian, gay, bisexual, transgender, intersexual and asexual (LGBTIA) persons are more likely to experience health issues such as depressive symptoms, anxiety and suicidal ideation throughout their lives^1,2^, and show higher rates of loneliness compared to their cis-heterosexual peers^3–6^. Loneliness, defined as disparity between one’s preferred state of social interactions and their actual state, contains two components: social loneliness, describing the absence of social connection and networks, and emotional loneliness, meaning the absence of a significant and close bond to others^7^. It is not restricted to old age and is affected by different socioeconomic factors as well as relationship quantity and quality^8,9^. There is evidence that for LGBTIA individuals, fewer social ties aggravate mental health burden and feelings of loneliness^10,11^.

Proximal and distal stressors unique to marginalized groups, as described in the Minority Stress Model^12^, help understand how health disparities in minority groups can occur. While disclosure of sexual orientation and gender identity, as well as social connectedness and familial support are deemed to have mitigating effects^13–15^, rejection from family members when disclosing sexual orientation and gender identity is not uncommon^16–18^. The relationship between LGBT adults and their parents has been described as emotionally weaker, with a decrease in kinship networks over time that is not compensated by other social contacts^19,20^. In comparison, data on the relationship of siblings with differing sexual identities indicate LGBT individuals to be more likely to disclose their sexual orientation first to siblings and friends, rather than to parents^21,22^. This indicates a sense of solidarity within these connections, though they are also described as potentially conflicting^23,24^. Compared to heterosexual relationships, LGBTIA are more open to alternative relationship configurations besides monogamous dynamics, and are less likely to be partnered^25,26^. Instead of relying primarily on family, support networks of LGBTIA often build on “chosen family”, i.e. the LGBT community and friends as close confidants^27–29^. During the COVID-19 pandemic, a shift in individual social networks from friends and colleagues to kin occurred in the general population^30^. Feelings of loneliness increased in Germany throughout all age groups in 2020, and while a decrease in the following year was observed, loneliness was still more prevalent than before the pandemic^31,32^.

While a body of research exists that focuses on the social support network of younger and older LGBTIA adults^10,11,15,33,34^ and the general population^8,9,30,35^, the differences in the social network between LGBTIA and non-LGBTIA persons, i.e. weekly contact to family members, friends and partners, and their implications for loneliness, have not been fully explored yet. Therefore, the first objective of this study was to examine the social network of LGBTIA persons and cis-heterosexual persons in Germany, focusing on differences in weekly contact with family, with friends and partner status. The second objective of the study was to investigate how these factors, alongside LGBTIA status, affected social and emotional loneliness. While the study took place during the COVID-19 pandemic when social distancing measures were in place^36^, this research article does not aim to examine the impact these interventions had on the social network of LGBTIA and cis-heterosexuals. Nonetheless, it acknowledges the impact of the pandemic on contact behavior.

## Methods

### Study design

We conducted an online survey on the living situation of German residents during COVID-19. The survey was accessible for two weeks in March and April 2020 (first data acquisition wave) and for three weeks in January and February 2021 (second data acquisition wave) and was created with “SoSci Survey”, a web-application that runs on a server located in Germany. In the two waves, data were collected independently from each other.

At the time, social distancing measures aiming to reduce transmission of COVID-19 were in place. In Germany, they encompassed a shutdown of public and private spaces as well as health-related services, and restricted individual social contact to household members and to one singular person of another household in public spaces^36^. Based on the same survey, data on depressive symptoms among asexual persons^37^ and LGBT persons^6^, as well as changes in health care utilization^38^ during COVID-19 have been published elsewhere.

### Data collection

The survey participants were recruited as convenience sample with a targeted oversampling of LGBTIA individuals. The online survey was shared through local newspapers, LGBTIA organizations and social media. All participants were informed about the content and the aims of the study as well as publication of the results prior to taking part and gave informed electronic consent. Participants did not receive any kind of incentive before or after completing the survey.

### Informed consent and ethics declaration

The study was conducted in accordance with the Declaration of Helsinki. All participants received the study information and gave informed consent online before participating in the survey. Since the questionnaire did not collect personalized data or any identifying information, in accordance with the professional regulations of the Berlin Medical Association (§ 15 Berufsordnung der Ärztekammer Berlin) and with German Law, we did not consult with an ethical review board.

### Measures

The survey contained questions on sociodemographic data (age, gender identity, sexual orientation, relationship status, living environment), on loneliness and on social contacts. Participants could choose gender identity and sexual orientation in multiple answer categories, in the first acquisition wave in one combined question (“Which of the following categories fit you best?”), and in the second data acquisition wave in two separate questions (“Which of the following categories regarding your sexual orientation/gender identity fit you best?”, with “aromantic” and “queer” as additional response options). Based on their selections, participants were split into categories: (1) LGBTIA (selection of at least one of the following categories: “aromantic”, “asexual”, “bisexual”, “homosexual”, “lesbian”, “pansexual “, “queer”, “gay”, “inter”, “non-binary”, “queer”, “trans”) and (2) cis-heterosexual (when none of the LGBTIA categories, but at least one of the categories “cis”, “woman”, “man”, “heterosexual” was selected). To account for the social network, we asked the participants about their relationship status and whether they had weekly contact with at least one parent, one sibling, and/or one friend at the time of participation, and about their weekly contact and relationship status in January 2020 in both survey waves. Contact could be either a meeting in person or a call. Regarding loneliness, the survey used the German version of the De Jong Gierveld Short Scale that contains items on emotional and social loneliness^39^. The answers of the Short Scale were coded on a 4-point Likert scale, with higher point values reflecting increased loneliness. A mean loneliness value of 2.5 was defined as cut-off for loneliness, in accordance to other studies^6,40^. Participants of the second survey wave were asked whether they had taken part in the survey before, but for data security reasons, we did not work with identification codes, and answers of the participants who responded in both waves were not individually matched.

### Statistical analyses

Statistical analysis was conducted with R Version 4.2.2^41^. To examine differences in social contacts between LGBTIA and cis-heterosexual participants, in each data acquisition wave we compared the number of participants who reported weekly contact to family, friends, and having a partner. Chi square tests were performed to examine difference in contacts between LGBTIA and cis-heterosexual participants. P-values lower than 0.05 were deemed statistically significant for our analyses.

To assess how LGBTIA status and the social network impacted social and emotional loneliness, we conducted linear regression analyses with social and emotional loneliness subscales of the De Jong Gierveld loneliness scale as dependent variables. In the first model, independent variables were LGBTIA status, living environment, age, data acquisition wave, and number and type of contact (face-to-face, phone call). In the second model, social contacts to parents, siblings, friends, as well as partner status were additional independent variables. The interaction between LGBTIA status and social network and living environment were added as interaction terms. Additionally, to account for potential differences in transgender participants in the LGBTIA group, we conducted a sensitivity analysis including only transgender and cis-heterosexual participants. The models were compared by R^2^.

### Missing data

Missing values for each variable are reported in Table 1. Missing values were not included in the percentages of the LGBTIA and cis-heterosexual group in Table 1. We performed no imputation, as the rate of overall missing values was under 5%^42^.

**Table 1:** Description of the sample by data acquisition wave and groups, *n* = 6784.

Group	First data acquisition wave (*n* = 2641)			Second data acquisition wave (*n* = 4143)		
	LGBTIA (*n* = 1880)	cis-heterosexual (*n* = 595)	Unassigned (*n* = 166)	LGBTIA (*n* = 3562)	cis-heterosexual (*n* = 440)	Unassigned (*n* = 141)
Age						
18 to 25 years	468 (24.9%)	180 (30.3%)	8	715 (20.1%)	55 (12.5%)	0
26 to 35 years	657 (35.0%)	207 (34.8%)	12	1137 (31.9%)	138 (31.4%)	0
36 to 45 years	367 (19.6%)	113 (19.0%)	23	820 (23.0%)	127 (28.9%)	0
46 to 55 years	242 (12.9%)	48 (8.1%)	15	582 (16.3%)	72 (16.4%)	1
56 years and older	143 (7.6%)	47 (7.9%)	9	307 (8.6%)	47 (10.7%)	0
Missing	3	0	99	1	1	140
Living environment^a^						
Rural community	141 (7.5%)	85 (14.3%)	11	305 (8.6%)	56 (12.7%)	0
Small-sized town	119 (6.3%)	69 (11.6%)	11	294 (8.3%)	60 (13.6%)	0
Middle-sized town	248 (13.2%)	134 (22.5%)	12	523 (14.7%)	77 (17.5%)	0
City	1359 (72.3%)	306 (51.4%)	33	2435 (68.4%)	244 (55.5%)	1
Missing	13	1	99	5	3	140
Gender identity^b^						
Man	446	114	0	1841	120	0
Woman	534	358	0	1182	314	0
Cis	372	57	0	1119	113	0
Trans	205	0	0	396	0	0
Non-binary	306	0	0	431	0	0
Inter	16	0	0	23	0	0
Queer (identity)^c^	–	–	–	841	0	0
Missing	0	0	99	0	0	116
Sexual Orientation^b^						
Heterosexual	37	480	0	103	427	0
Gay (German Slang)	479	0	0	1042	0	0
Lesbian	442	0	0	701	0	0
Bisexual	368	0	0	601	0	0
Queer (orientation)^†††^	–	–	0	949	0	0
Pansexual	310	0	0	427	0	0
Asexual	145	0	0	300	0	0
Aromantic ^d^	–	–	0	118	0	0
Missing	0	0	99	0	0	116
Loneliness score (mean, [standard deviation])						
Total	2.30 [0.61]	2.00 [0.56]	–	2.39 [0.60]	2.19 [0.57]	–
Social	1.95 [0.73]	1.64 [0.66]	–	2.04 [0.73]	1.82 [0.69]	–
Emotional	2.64 [0.72]	2.37 [0.69]	–	2.75 [0.70]	2.56 [0.69]	–

^a^Rural community: up to 5000 inhabitants, small-sized town: >5000–20000 inhabitants; middle-sized town > 20000–100000 inhabitants, urban environment: >100000 inhabitants.

^b^Gender Identity, Sexual Orientation: multiple answers possible.

^c^Queer as both gender identity and sexual orientation was added in the second wave of data acquisition.

^d^Aromantic as a sexual orientation was added in the second wave of data acquisition.

*LGBTIA* lesbian, gay, bisexual, transgender, intersex, asexual.

## Results

### Description of the sample in the two data acquisition waves

6784 persons participated in the study giving informed consent, with 2641 in the first and 4143 in the second data acquisition wave. 108 participants of the second data acquisition wave stated that they had already answered the survey in the first data acquisition wave, 743 could not remember. 3139 had not participated before, and 153 did not answer the question. The majority of the respondents was between 18 and 35 years old (60.3% in the first data acquisition wave, 51.1% in the second data acquisition wave), and a small percentage was 66 years and older (2.3% and 1.6% respectively; cf. Table 1). Approximately two thirds of respondents (first data acquisition wave: 67.2%, second data acquisition wave: 67.1%) lived in a city with 100,000 inhabitants or more.

### Description of the LGBTIA and cis-heterosexual sample

In total, 5442 participants identified as LGBTIA, 1035 people as cis-heterosexual. In 307 cases (166 in the first and 141 in the second data acquisition wave), respondents could not be assigned to any group because data on gender identity and sexual orientation were missing. In each of the categories on gender identity and sexual orientation, at least 400 participants were represented, except for “inter” (*n* = 39) and “aromantic” (*n* = 118). Over two thirds (69.9%) of LGBTIA people lived in a city with 100,000 or more inhabitants, while this only applied to half (53.3%) of cis-heterosexuals. LGBTIA participants rated higher than cis-heterosexuals both on the social and the emotional loneliness score. Table 1 offers a description of the sample by data acquisition wave and group.

### Weekly social contact in January 2020

**Fig. 1:**
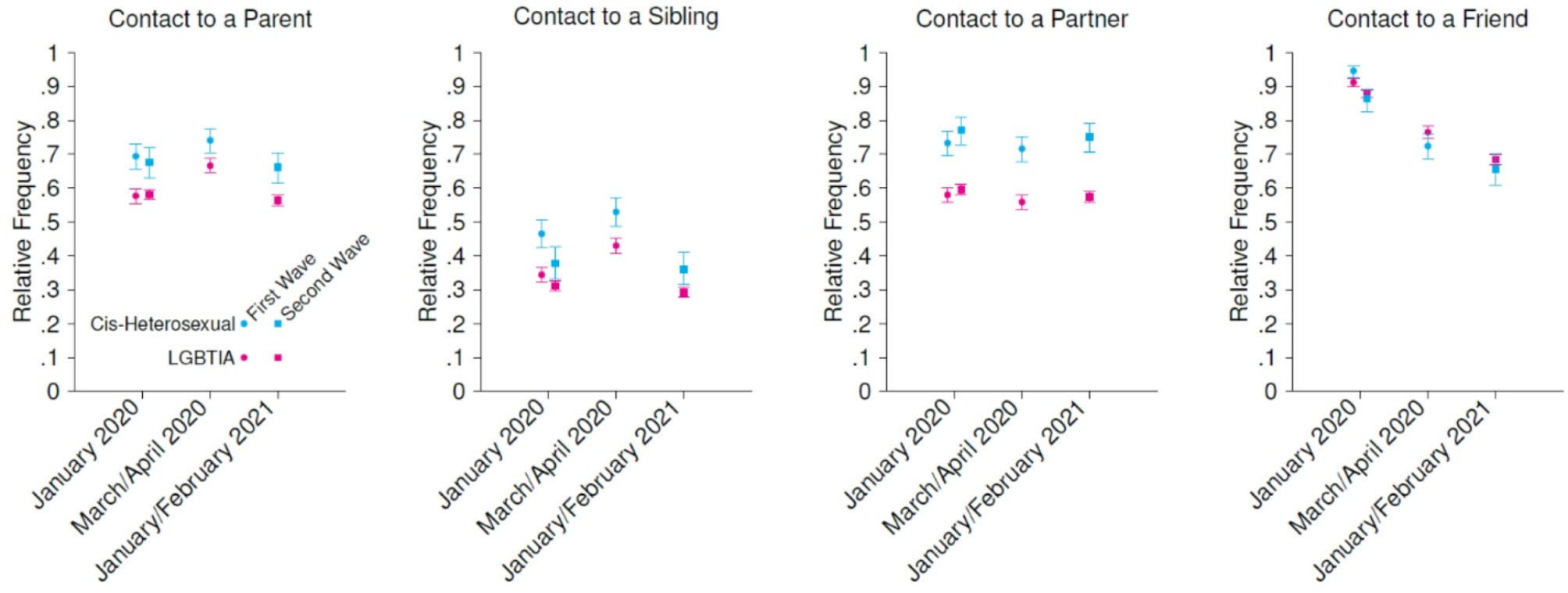
Relative frequency of contact to parents, siblings, partners, and friends in the LGBTIA and cis-heterosexual group in the first and second wave of data acquisition.

Figure 1 shows the relative frequency of contacts by wave and by group. Weekly contact to parents and siblings differed significantly between the LGBTIA and cis-heterosexual participants: In both waves of data acquisition, when asked about weekly contact in January 2020, more cis-heterosexual than LGBTIA participants reported to have had contact at least once a week to at least one parent (69.4% vs. 57.7% in the first wave, 67.6% vs. 58.1% in the second wave), and at least one sibling respectively (46.5% vs. 34.4% in the first wave, 37.8% vs. 31.8% in the second wave). A significant difference in contact to friends in January 2020 between the LGBTIA and cis-heterosexual participants was shown in the first wave of data acquisition. Similarly, in both waves, more cis-heterosexual than LGBTIA participants reported that they had a partner in January 2020. The difference between the two groups regarding partnerships was significant in both waves (see Table 2 for χ^2^ and p-values of all categories).

**Table 2:** Social network (contact with at least one parent, one sibling or one friend, partner status) in January 2020 and at the time of data acquisition.

	First data acquisition wave (Mar/Apr 2020)				Second data acquisition wave (Jan/Feb 2021)			
	LGBTIA	cis-heterosexual	χ^2^	p	LGBTIA	cis-heterosexual	χ^2^	p
“I have contact with at least one parent at least once a week.”								
Jan 2020	966 (57.7%) [55.3, 60.0]	366 (69.4%) [65.4, 73.2]	22.8	< 0.001	1852 (58.1%) [56.4, 59.8]	259 (67.6%) [62.8, 72.1]	12.4	< 0.001
Time of Data Acq.	1117 (66.6%) [64.3, 68.8]	398 (74.1%) [70.2, 77.6]	10.3	< 0.001	1801 (56.4%) [54.6, 58.1]	255 (66.1%) [61.2, 70.6]	12.8	< 0.001
“I have contact with at least one sibling at least once a week.”								
Jan 2020	561 (34.4%) [32.1, 36.7]	237 (46.5%) [42.2, 50.8]	23.7	< 0.001	968 (31.1%) [29.5, 32.8]	137 (37.8%) [33.0, 42.9]	44.7	0.01
Time of Data Acq.	709 (43.0%) [40.6, 54.4]	277 (53.0%) [48.7, 57.2]	15.6	< 0.001	910 (29.2%) [27.7, 30.9]	133 (36.0%) [31.3, 41.1]	7.0	0.008
“I have a partner.“								
Jan 2020	980 (58.0%) [55.6, 60.3]	385 (73.3%) [69.4, 76.9]	39.2	< 0.001	1920 (59.5%) [57.8, 61.2]	300 (77.1%) [72.7, 81.0]	44.7	< 0.001
Time of Data Acq.	946 (55.9%) [53.5, 58.2]	385 (71.6%) [67.6, 75.2]	41.1	< 0.001	1872 (57.5%) [55.8, 59.2]	298 (75.1%) [70.6, 79.1]	44.5	< 0.001
“I have contact with at least one friend at least once a week.”								
Jan 2020	1571 (91.2%) [89.8, 92.5]	521 (94.6%) [92.3, 96.2]	5.8	0.02	2875 (87.8%) [86.6, 88.9]	340 (86.3%) [82.5, 89.3]	0.62	0.43
Time of Data Acq.	1306 (76.5%) [74.4, 78.4]	393 (72.4%) [68.5, 76.0]	3.5	0.06	2217 (68.4%) [66.8, 70.0]	252 (65.6%) [60.7, 70.2]	1.1	0.3

*Acq.* Acquisition.

*LGBTIA* lesbian, gay, bisexual, transgender, intersex, asexual.

### Weekly social contact in March/April 2020 and January/February 2021

Both in March/April 2020 and in January/February 2021, more cis-heterosexuals than LGBTIA participants had contact with at least one parent and at least one sibling, respectively, and more cis-heterosexual participants had a partner. The difference between the LGBTIA and the cis-heterosexual groups in contact to parents and siblings, as well as having a partner, was statistically significant.

### Weekly social contact and its relation to loneliness

As seen in Table 1, LGBTIA participants showed higher mean values on the loneliness scale than cis-heterosexual participants. Table 3 shows the results of the regression analysis. In the regression model that did not include social network factors, identifying as LGBTIA was associated with increased social and emotional loneliness. In the regression analysis that included social network variables, the influence of being LGBTIA on both social and emotional loneliness decreased, though the decrease in emotional loneliness was less pronounced. The models with social networks had a better explanation power as highlighted by the increased R^2^ value. Several factors contributed to social loneliness: Having no partner, lack of contact with parents, siblings or friends, and older age were all associated with higher levels of social loneliness. While identifying as LGBTIA had no significant impact on social loneliness on its own, there was a positive interaction when participants identified as LGBTIA and did not have a partner in combination, meaning LGBTIA without partner had higher risk for social loneliness. A larger number of actual contacts reduced the risk for social loneliness. Neither the survey wave, nor the living environment had an impact social loneliness. The strongest contributors to social loneliness was having no contact with friends and the combination of LGBTIA status and being without a partner.

**Table 3:** Regression analysis: changes in social and emotional loneliness on the de Jong Gierveld short scale.

Variable	Coefficient [95% CI]			
	Without social network variables	With social network variables	Without social network variables	With social network variables
	Social loneliness	Social loneliness	Emotional loneliness	Emotional loneliness
(Intercept)	1.84 [1.69, 1.99]	1.65 [1.49, 1.81]	2.55 [2.4, 2.69]	2.3 [2.14, 2.46]
LGBTIA vs. cis-heterosexual	0.27 [0.12, 0.42]	0.11 [− 0.06, 0.28]	0.28 [0.14, 0.43]	0.22 [0.06, 0.38]
Having no partner		0.11 [0.01, 0.21]		0.33 [0.23, 0.43]
Having no partner * LGBTIA		0.19 [0.08, 0.31]		0.11 [0, 0.22]
No contact to friends		0.22 [0.12, 0.33]		0.07 [− 0.03, 0.18]
No contact to friends * LGBTIA		0.09 [− 0.02, 0.21]		0 [− 0.11, 0.11]
No contact to parents		0.12 [0.01, 0.24]		0.08 [− 0.03, 0.19]
No contact to parents * LGBTIA		− 0.01 [− 0.13, 0.11]		− 0.01 [− 0.12, 0.11]
No contact to siblings		0.13 [0.03, 0.23]		0.05 [− 0.05, 0.14]
No contact to siblings * LGBTIA		− 0.03 [− 0.14, 0.08]		− 0.03 [− 0.14, 0.08]
Survey wave	0.08 [0.04, 0.12]	0.04 [0, 0.08]	0.16 [0.12, 0.2]	0.15 [0.11, 0.19]
Living environment: small-sized town	0.07 [− 0.12, 0.26]	0.07 [− 0.12, 0.26]	0.017 [0–0.17, 0.2]	0.11 [− 0.08, 0.3]
Living environment: middle-sized town	0.07 [− 0.1, 0.23]	0.01 [− 0.15, 0.18]	0.1 [− 0.06, 0.27]	0.11 [− 0.06, 0.27]
Living environment: urban	− 0.001 [− 0.15, 0.14]	− 0.03 [− 0.18, 0.11]	0.07 [− 0.07, 0.21]	0.09 [− 0.05, 0.23]
Small-sized town * LGBTIA	− 0.05 [− 0.26, 0.17]	− 0.05 [− 0.26, 0.17]	− 0.03 [− 0.24, 0.18]	− 0.13 [− 0.35, 0.08]
Middle-sized town * LGBTIA	− 0.02 [− 0.21, 0,17]	0.01 [− 0.15, 0.18]	− 0.08 [− 0.26, 0.12]	0.11 [− 0.06, 0.27]
Urban * LGBTIA	− 0.06 [− 0.22, 0.11]	− 0.03 [− 0.18, 0.11]	− 0.81 [− 0.24, 0.08]	0.09 [− 0.05, 0.23]
Age	0,036 [0.02, 0.05]	0.03 [0.01, 0.04]	− 0.09 [− 0.1, − 0.1]	− 0.07 [− 0.09, − 0.06]
Personal/direct contact	− 0.03 [− 0.03, − 0.02]	− 0.02 [− 0.02, − 0.02]	− 0.01 [− 0.01, − 0.01]	− 0.01 [− 0.01, 0]
Contact by video/phone call	− 0.04 [− 0.04, − 0.03]	− 0.02 [− 0.03, − 0.02]	− 0.03 [− 0.03, − 0.02]	− 0.02 [− 0.03, − 0.01]
R-squared	0.09	0.18	0.07	0.16

Regarding emotional loneliness, being without a partner, identifying as LGBTIA, younger age and the wave of data acquisition were associated with emotional loneliness. The combination of being LGBTIA and having no partner aggravated the effect on emotional loneliness. A larger number of actual contacts was negatively associated with emotional loneliness. Unlike social loneliness, contact with friends, parents and siblings, as well as the living environment, were not related to emotional loneliness.

The sensitivity analysis that included only participants identifying as transgender and cis-heterosexual in the regression models revealed no major differences. The weight of the coefficient of being LGBT was slightly more pronounced in this sensitivity analysis.

## Discussion

Addressing our research questions, our results show that weekly contact with parents and siblings was consistently lower in the LGBTIA group than in the cis-heterosexual group, and that they were significantly less likely to have a partner in both acquisition waves. Weekly contact to friends was similar in both groups. The social network contributed to a reduction in feelings of loneliness: social loneliness was higher when participants did not have contact with family members or with friends. Being LGBTIA and being without a partner in combination had the highest effect on emotional loneliness. A higher number of actual contacts lowered both social and emotional loneliness. Social loneliness increased with age, while younger age was associated with emotional loneliness. The differences in social network that we observed partly explained a higher risk for social loneliness of LGBTIA persons and, to a lower degree, the higher risk for emotional loneliness.

Snapp et al. reported family acceptance as the strongest predictor for well-being in LGBT youth^15^. McConnell et al. describe social support and mental health trajectories in LGBT youth, suggesting that a lack of familial support in LGBT youth is associated with a higher risk for mental health disparities in adulthood, but an increase in family support was observed over time^13^. While our results do not allow us to evaluate the quality of social contacts to parents and siblings, they confirm that in our participant group, contact frequency to parents and siblings was significantly lower in the LGBTIA group. A moderately lower level of emotional closeness, a tendency for higher rates in parent-child conflicts and reduced contact to parents have been described for LGBT^19^. A study from 2016 suggests that LGBT older adults have less weekly contact to parents and live at a greater distance from them than cis-heterosexuals, resulting in weaker kinship networks that are not compensated when taking into account other forms of social support like companionship networks or community networks^20^. In our results, weekly contact to family was lower for LGBTIA than for cis-heterosexuals, and only affecting social loneliness, possibly indicating that contact to family is balanced out by other contacts to friends and partners. The changes we observed in the LGBTIA and the cis-heterosexual groups are consistent with other studies that describe a shift in social network structure towards family in the general population^30^.

When asked about whom they would rely on in times of need, LGBTIA persons in other studies responded with preferring friends, rather than kin, for social support and connections^27,29^. The importance of friends in LGBTIA persons is reflected in our results as well: it is the only group in which LGBTIA participants and cis-heterosexual participants show similar weekly contact. While our regression analysis did not show a significant relation between face-to-face contact and loneliness, a study on German LGBT people found face-to-face contact, as opposed to contact through digital means, to be positively associated with reducing feelings of loneliness and mitigating mental health burden during the COVID-19 pandemic^10^, coming to the conclusion that social connectedness should be promoted particularly in communities, as community involvement has been associated with lower levels of loneliness^4^. This is further underlined by another study that highlighted the positive association between social networks comprised specifically of other LGBTIA persons and health outcomes, reduced stress and less feelings of discrimination in the LGBTIA population^11^.

Having a partner had the strongest influence on emotional loneliness in the regression analysis both for LGBTIA and cis-heterosexual participants. In accordance to our findings, other studies point at LGBTIA persons to be less often in relationships^26^, and be less likely to live together with their partner: for example, a study on a German LGB population found a difference of 10% points between cis-heterosexuals and LGB individuals regarding relationship status and a 12% point difference in coresidential relationships^29^. Non-monogamous relationship configurations seem to be more common in the LGBTIA community^25^, which might have been difficult to maintain during the COVID-19 pandemic when our survey took place.

Our research has several strengths and limitations. Our study adds to the existing body of research by providing further insight in the relation of social network structures and feelings of loneliness in an LGBTIA population, the impact of the social network on both social and emotional loneliness, emphasizing the importance of partnerships as a risk factor for loneliness. Our study design was limited to a German population, and results could differ in other countries with differing societal and judicial acceptance of sexual and gender minorities. Through targeted oversampling, we managed to gather a large number of LGBTIA participants which is otherwise hard to sample, but the representativeness of the sample for the general population might be limited. For example, compared to the representative GEDA study of health in the German population, in which approximately 41% were 18–44 years old and 38.5% lived in an area with 100,000 inhabitants or more^43^, our sample was younger (76.9% between 18 and 44 years) and more likely to live in an urban environment. Loneliness in rural areas, especially for sexual and gender diverse persons and older adults, might be more pronounced than in our results. While the LGBTIA and non-LGBTIA groups were similar in age distribution, there were differences in the living environment, potentially representing confounders for contact with family or friends.

Other than sensitivity analysis on transgender participants, we did not conduct subgroups analyses on differences in weekly contact for all sexual and gender identities included in the LGBTIA acronym due to sample sizes. However, we acknowledge that these subgroups may differ regarding feelings of loneliness, as pointed out in another study^6^.

In a German study using longitudinal data from a general population sample, an increase in depressive symptoms and loneliness was described in the 12-month follow-up after the COVID-19 outbreak^32^. In our study, 108 participants reported to have participated in both waves of the survey and 743 did not remember if they had participated in the first wave of the survey. Since we did not use identifying information or identification codes in the survey, we were unable to match their responses to highlight individual changes over time. Furthermore, a selection bias cannot be ruled out: through sampling online, digital literacy was a requirement to participate. There is a possibility that, due to sharing the survey via LGBTIA organizations, people that were already socially and communally connected, took part in the survey, therefore the frequency of contact with friends and family could be lower in the entire LGBTIA population than it was in our sample.

Given that the second data wave of data acquisition occurred in January/February 2021 and participants were asked about their contacts both at the time of the survey wave and in January 2020, there is a possibility of recall bias regarding their reports of contacts from January 2020. However, since the proportion of participants in contact with parents and friends in January 2020 are similar in both waves of data acquisition, we deem this bias to be of limited importance.

## Conclusion

Regarding the association of the social network and loneliness, on an individual level, we encourage health care professionals to consider sexual orientation and gender identity of their patients and raise awareness for feelings of loneliness and other associated health problems. In accordance with our results, it is especially important to emphasize on those without a partner when inquiring about LGBTIA status. On a communal level, current intervention formats such as social prescribing should be investigated regarding their effects on mitigating loneliness in the LGBTIA population. Further research can build upon our findings regarding differences in social networks in LGBTIA and cis-heterosexual persons, exploring the long-term dynamics of the components of the social network, how they change over time and how they affect feelings of loneliness.

## Data Availability

The datasets used and analysed during the current study are available from the corresponding author on reasonable request.
